# Enabling People With Intellectual and Sensory Disabilities to Trigger a Tablet’s Delivery of Task Instructions by Walking to the Tablet: Proof-of-Concept Study

**DOI:** 10.2196/59315

**Published:** 2024-06-12

**Authors:** Giulio E Lancioni, Nirbhay N Singh, Mark F O’Reilly, Jeff Sigafoos, Gloria Alberti, Isabella Orlando, Valeria Chiariello, Lorenzo Desideri

**Affiliations:** 1 Lega F D’Oro Research Center Osimo Italy; 2 Department of Psychiatry and Health Behavior Augusta University Augusta, GA United States; 3 College of Education University of Texas at Austin Austin, TX United States; 4 School of Education Victoria University of Wellington Wellington New Zealand; 5 Department of Psychology Sigmund Freud University Milan Italy

**Keywords:** technology, tablet, task, instructions, intellectual disability, visual impairment, hearing impairment

## Abstract

**Background:**

People with intellectual and sensory or sensory-motor disabilities tend to have problems performing multistep tasks. To alleviate their problems, technological solutions have been developed that provide task-step instructions. Instructions are generally delivered at people’s request (eg, as they touch an area of a computer or tablet screen) or automatically, at preset intervals.

**Objective:**

This study carried out a preliminary assessment of a new tablet-based technology system that presented task-step instructions when participants with intellectual and sensory disabilities walked close to the tablet (ie, did not require participants to perform fine motor responses on the tablet screen).

**Methods:**

The system entailed a tablet and a wireless camera and was programmed to present instructions when participants approached the tablet, that is, when the camera positioned in front of the tablet detected them. Two instructions were available for each task step. One instruction concerned the object(s) that the participants were to collect, and the other instruction concerned the “where” and “how” the object(s) collected would need to be used. For 3 of the six participants, the two instructions were presented in succession, with the second instruction presented once the required object(s) had been collected. For the other 3 participants, the two instructions were presented simultaneously. Instructions consisted of pictorial representations combined with brief verbal phrases. The impact of the system was assessed for each of the 2 groups of participants using a nonconcurrent multiple baseline design across individuals.

**Results:**

All participants were successful in using the system. Their mean frequency of correct task steps was close to or above 11.5 for tasks including 12 steps. Their level of correct performance tended to be much lower during the baseline phase when they were to receive the task-step instructions from a regular tablet through scrolling responses.

**Conclusions:**

The findings, which need to be interpreted with caution given the preliminary nature of the study, suggest that the new tablet-based technology system might be useful for helping people with intellectual and sensory disabilities perform multistep tasks.

## Introduction

### Background

People with intellectual disabilities tend to have problems carrying out multistep tasks, largely due to difficulties in remembering the different steps included in the tasks and the order in which they should be performed [1-5]. The problems may be even greater in situations where intellectual disabilities are combined with sensory or sensory-motor impairments [6-8]. In spite of the difficulties encountered, fostering the ability to carry out multistep tasks remains a main rehabilitation objective, vital for ensuring that people will be able to achieve functional occupation and have a constructive role within their daily contexts and possibly within vocational contexts [1,9-12]. Such achievement is considered critical for advancing their condition, offering them new socially adaptive opportunities, and improving their quality of life [8,11,13-19].

Given the relevance of enabling people to manage the performance of multistep tasks, a large variety of studies have been conducted with the aim of reaching this goal with the support of technological solutions [1,4,20]. These technological solutions, designed to provide instructions for performing task steps correctly and in the right sequence, present several differences [10,21]. The most obvious differences concern (1) the characteristics of the instructions provided (eg, static pictorial images vs video clips illustrating the steps with or without an accompanying verbal phrase describing the steps) and (2) the way those instructions are made available [1,4].

With regard to the latter aspect (ie, the way instructions are made available), two main approaches can be pointed out. The first approach relies on the use of computer or tablet devices that present instructions for the task steps based on participants’ requests. Typically, participants initiate the request by performing a specific action such as touching an area of the computer or tablet screen [5,9,22-24]. The second approach relies on computer, tablet, or smartphone devices presenting the instructions automatically, at preset time intervals, eliminating the need for participants to produce specific request responses [7,25,26]. The intervals between instructions are decided by staff personnel familiar with the participants and the time they require for carrying out the different task steps.

The second approach may be considered advantageous for participants who cannot successfully use the first approach due to challenges in providing appropriate responses on computer or tablet screens (eg, inaccuracy in executing touch and scroll responses required to operate these devices) [27,28]. On the other hand, the presentation of instructions at preset time intervals may not always be consistent (in synchrony) with the participants’ performance. Although staff may have estimates of the times required by the participants for carrying out the task steps, the participants’ response speed and efficacy may fluctuate within and across days, making the intervals programmed based on those estimates too long or too short [8,16]. This may lead to participants missing some instructions and related task steps or having to wait for the instructions.

A possible way to bypass the shortcomings of the aforementioned approaches may involve the development of a technology system that (1) presents instructions without requiring the participants’ performance of fine motor responses on the computer or tablet screen and simply (2) associates instruction presentation with participants’ walking toward the system [8,16,27]. Such a system would ensure that participants who struggle with performing accurate motor responses on a computer or tablet screen do not need to use those responses. At the same time, this system would guarantee that instructions are delivered at the appropriate time (directly linked to people’s actions) rather than at preset time intervals [8,16,29].

### Objectives

This study aimed to set up such a system and carry out a preliminary evaluation of it with 6 participants with intellectual and sensory disabilities. The system consisted of a tablet and a wireless camera and was programmed to present instructions when the participant approached the tablet, that is, as the participant was spotted by the camera positioned in front of the tablet. Two instructions were available for each task step. One instruction concerned the object(s) that the participants were to collect, and the other concerned the “where” and “how” the collected object(s) were to be used. For 3 participants, the two instructions were presented in succession, with the second instruction displayed after the required object(s) had been collected. For the other 3 participants, both instructions were presented simultaneously. Instructions consisted of pictorial representations combined with brief verbal phrases. For each of the two groups of participants, the study was conducted following single-case research methodology.

## Methods

### Participants

Table 1 lists the participants included in the study (categorized into two groups of 3 based on their use of the task-step instructions) and reports their chronological ages and their Vineland age equivalents for daily living skills (personal subdomain) and receptive communication. The participants, who have pseudonyms (Table 1), were between 23 and 62 years of age. All of them were diagnosed with sensory disabilities. Specifically, Allie had severe hearing loss. Sylvie, Rowan, Demi, and Jolene had serious impairments of their neurovisual system, leading to severe limitations in their visual acuity. Emory presented with severe limitations in her visual acuity as well as severe hearing loss. The use of eyeglasses allowed all participants to discriminate pictorial images of familiar objects on a tablet screen and to navigate easily within familiar contexts. Vineland age equivalents (measured via the second edition of the Vineland Adaptive Behavior Scales [30,31]) ranged from 4 years to 5 years and 3 months for personal daily living skills and from 3 years and 4 months to 4 years and 3 months for receptive communication. All participants attended rehabilitation and care centers, where the psychological services classified their level of functioning within the moderate intellectual disability range. However, no IQ scores were available.

The participants were recruited for the study based on a number of general criteria. First, they were unable to carry out multistep tasks without staff guidance or specific step instructions. Second, they could use pictorial representations alone or in combination with simple verbal phrases as instructions for the performance of task steps. Third, they expressed their willingness to use the technology system adopted in this study (and shown to them in advance) for carrying out multistep tasks involving familiar material and areas within their daily contexts. Fourth, they had poor fine motor skills and were considered unable to reliably use a tablet for accessing a series of task-step instructions. Fifth, staff supported their involvement in the study and considered technology-aided task engagement a positive goal for the participants and their contexts.

**Table 1:** Participants’ chronological age and Vineland age equivalents for daily living skills (personal subdomain) and receptive communication.

Participants (pseudonyms)		Chronological age (years)	Vineland age equivalents^a^ (years, months)	
			Daily living skills (personal subdomain)	Receptive communication
**First group**				
	Rowan	23	4, 2	3, 4
	Allie	62	5, 3	3, 11
	Sylvie	48	4, 0	3, 4
**Second group**				
	Jolene	48	4, 4	4, 3
	Emory	61	5, 1	3, 11
	Demi	49	5, 1	4, 3

^a^Age equivalents are based on the Italian standardization of the Vineland scales [30].

### Ethical Considerations

The study was approved by the Ethics Committee of the Lega F. D’Oro, Osimo (Ancona), Italy (P072820235). All procedures performed were in accordance with the ethical standards of the institutional and national research committee and with the 1964 Helsinki Declaration and its later amendments or comparable ethical standards.

As mentioned above, the participants had expressed their willingness to use the technology system to carry out tasks involving familiar material. Moreover, staff had indicated that the participants would enjoy performing the tasks provided that difficulties and errors (and thus frustration) would be largely avoided, which was the expectation within this study. While these two points suggested the study would be a positive experience for the participants, it was not possible for them to read and sign a formal consent document. Consequently, their legal representatives were directly involved in the consent process, reading and signing the consent forms on the participants’ behalf.

### Setting, Sessions, Tasks, Instructions, and Research Assistants

Familiar rooms within the participants’ daily environments constituted the setting for the study. Sessions were typically carried out 1 or 2 times per day, 4 to 6 days a week. During each session, the participants were asked to perform 1 task. Tasks consisted of combinations of 12 steps. Each step involved 2 simple actions, which were familiar and meaningful to the participants, for example, “take the toilet paper” and “bring the toilet paper to the men’s room.” The combinations of steps (and related actions) led to a recognizable and practically relevant outcome, such as setting up a bathroom and cleaning the entrance, arranging the living room and putting away papers and books, and preparing or cleaning the dining room [16]. Tasks could be flexible, that is, they could include different combinations of steps on different days based on practical and environmental conditions [16]. Moreover, a number of steps could be used across different tasks. In total, 9 tasks were available to each participant. Textbox 1 provides a combination of 12 steps that could be included in a task such as supplying the bathroom and arranging the kitchen.

**Figure 1:**
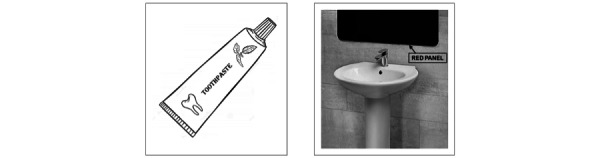
The 2 pictures represent the actions of collecting the toothpaste and bringing it to the washbasin of the red bathroom.

**Figure 2:**
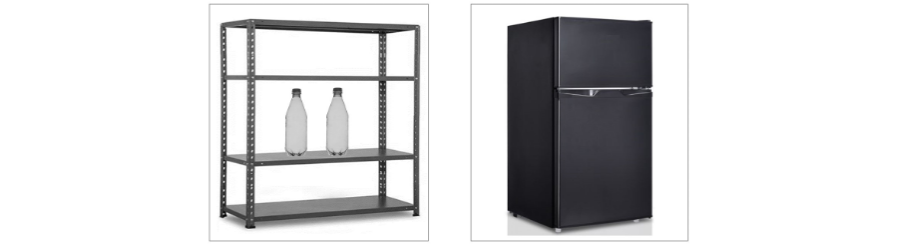
The 2 pictures represent the actions of collecting 2 bottles from a shelf and putting them in the refrigerator.

The instructions the tablet provided for the 2 actions involved in each task step consisted of 2 pictures (Figure 1 and Figure 2) accompanied by brief verbal phrases (explained further under the *Technology System* section below). For the first 3 participants listed in Table 1 (ie, Rowan, Allie, and Sylvie), the 2 pictures were presented separately (ie, one at a time in sequence), and each picture was accompanied by a verbal phrase matching it. For the other 3 participants (ie, Jolene, Emory, and Demi), the 2 pictures were presented simultaneously (ie, one next to the other, as shown in Figure 1 and Figure 2), accompanied by a verbal phrase matching them (explained under the *Technology System* section below).

The presentation of the two instructions available for each task step in sequence or simultaneously was based on the participants’ history, that is, their use of the pictures within their daily contexts, under the supervision of regular staff personnel. The research assistants were 4 women who held a master’s degree in psychology and had experience with the implementation of technology-aided programs with people with intellectual and multiple disabilities as well as with data collection strategies.

**A combination of 12 steps for supplying the bathroom and arranging the kitchen.**

- Take the toilet paper and bring it to the men’s bathroom.
- Take the towel and bring it to the ladies’ bathroom.
- Take the toothpaste and bring it to the men’s bathroom.
- Take the toilet paper and bring it to the ladies’ bathroom.
- Take the deodorant and bring it to the ladies’ bathroom.
- Take liquid soap and bring it to the men’s bathroom.
- Take the aluminum and bring it to the microwave.
- Take paper towels and put them in the kitchen drawer.
- Take the chips and put them on the kitchen table.
- Take the flowers and put them in the kitchen sink.

### Technology System

#### Basic Components

The technology included (1) a Samsung Galaxy tablet with an internet connection and MacroDroid and CloudEdge apps and (2) a DEATTI wireless (battery-powered) camera with a passive infrared sensor [32]. The tablet was also fitted with (1) pictures and verbal phrases used as instructions for the task steps; (2) positive-feedback pictures and praise words shown after the completion of each task step; and (3) videos with the participants’ preferred music, comic sketches, or food preparation presented after the completion of the last task step. The tablet was located in one of the rooms used for the tasks. The camera was positioned about 1.5 meters before the tablet. By walking to the tablet, the participants automatically activated the camera, making it send an input to the tablet via the CloudEdge app. This input was used by the MacroDroid app to make the tablet present task-step instructions.

#### Instructions Presentation

The first 3 participants (ie, Rowan, Allie, and Sylvie) received the two instructions available for each task step in succession (explained in the *Setting, Sessions, Tasks, Instructions, and Research Assistants* section). With a task step such as “bringing liquid soap from a store cabinet to the sink area of a specific bathroom,” for example, the instruction the participants received the first time they approached the tablet consisted of a picture showing the liquid soap inside a store cabinet (or simply the liquid soap) accompanied by the verbal phrase “take the soap.” The instruction they received the second time they approached the tablet for that step (while they were carrying the soap they had collected from the cabinet) involved a picture representing the soap on the sink of the red bathroom accompanied by the verbal phrase “bring the soap to the red bathroom.” Once a step was completed, approaching the tablet led to the tablet’s presentation of (1) positive feedback with a picture showing hand clapping, thumbs up, or another representation indicating approval and a praise word, and (2) the first instruction for the following task step. The process continued as described above for all other steps of the task and included the presentation of a 2.5-minute video of a preferred (music, comic, or food preparation) event following the completion of the last step. After the delivery of an instruction, the system had a brief period (15-25 seconds) of inertia to ensure that the participant could go back for a second look at the tablet screen without a change of instruction.

For the last 3 participants (ie, Jolene, Emory, and Demi), the tablet presented the two instructions available for each task step simultaneously. For example, for a step such as “bringing liquid soap from a store cabinet to the sink area of the red bathroom,” the tablet presented a picture showing soap (or soap in the cabinet) to the left and a picture showing soap on the sink of the red bathroom to the right and accompanied such presentation with a phrase like “take the soap and bring it to the red bathroom.” Returning to the tablet (ie, after completing a step) triggered the tablet’s presentation of positive feedback plus praise word followed by the presentation of the instructions for the next task step. The positive feedback and praise word after each completed step, the video of a preferred event at the end of the task, and the idleness of the tablet after the delivery of instructions matched those used for the first 3 participants.

### Experimental Conditions and Data Analysis

The study started with a pretest verifying whether the participants could carry out the tasks independent of specific step instructions. After the pretest, each of the two groups of participants had a baseline phase followed by an intervention phase. These phases were implemented according to a nonconcurrent multiple baseline design across participants [33,34]. In practice, the participants of each group received different numbers of baseline sessions before the start of the intervention with the technology system. Pretest, baseline, and intervention sessions were implemented by the research assistants. To make sure that their application of the procedural conditions was accurate (that their level of procedural fidelity was high), two strategies were adopted. One involved their preliminary familiarization with those conditions while the other involved regular feedback on their performance [35]. Feedback was delivered by a research coordinator who had access to video recordings of the sessions.

The participants’ data concerning the correctly performed task steps were reported in graphic form. To simplify the graphic presentation, data points were made to represent blocks of sessions. The baseline and intervention frequencies of correct task steps were compared using the “Percentage of data points Exceeding the Median” method [36,37]. This method, which is one of the most practical tools to evaluate single-case research data, served to determine how many data points of the intervention phase were above the baseline median.

### Pretest

The pretest included 5 sessions. Each session started with the research assistant asking the participants to carry out a task. The request was made via a simple verbal statement and a general pictorial representation. The statement summarized what the participants were to do (eg, “you can supply the bathroom and set up the kitchen table”). The pictorial representation included a drawing of the areas (bathroom and kitchen table) involved in the task. The research assistant did not intervene if the participants carried out steps involved in the task. If the participants remained passive for 30-60 seconds or carried out a step not involved in the task, the research assistant provided guidance for a task step (eg, helped them to bring the toilet paper to a red bathroom). The session continued until the participants had carried out all task steps or had received the research assistant’s guidance for the performance of 2 steps. All the steps omitted as well as those carried out with the research assistant’s guidance were counted as noncorrect. At the end of a session, the participants were presented with a 2.5-minute video of preferred music, comic, or food preparation events.

### Baseline

The baseline included 7, 8, and 13 sessions for the participants of the first group and 6, 8, and 12 sessions for the participants of the second group. Those sessions served to determine whether the participants were able to use a tablet independently to obtain task-step instructions and then carry out those steps. Each session started with the research assistant placing a tablet on a desk and asking the participants to use it to get instructions for a specific task. Meanwhile, the research assistant demonstrated how to use the tablet (ie, operating horizontal scrolling) to receive the step instructions. If participants were unsuccessful or passive for 30-60 seconds, the research assistant provided guidance (ie, carried out the tablet scrolling for them and ensured that they performed the task step indicated by the tablet instructions). Two instances of guidance from research assistants were allowed per session. A session lasted until the participants had either carried out the last step of the task or failed to progress (eg, due to a new unsuccessful or passive period following the research assistant’s guidance instances or due to inaccurate scrolling leading them to skip the instructions or shut the presentation process). At the end of a session, the participants were presented with a 2.5-minute video of their preferred music, comic, or food preparation events.

### Intervention

The intervention phase included 97, 83, and 88 sessions for the participants of the first group and 87, 64, and 69 sessions for the participants of the second group. During the intervention, the participants had the technology system that worked as described in the *Technology System* section. The objective was to determine whether the system was suitable to help the participants carry out the tasks correctly. Each session started with the research assistant accompanying the participants to the area where the tablet was available (ie, just before the camera). When the camera detected the participants, the tablet was triggered to produce the first instruction delivery. All the rest was as described in the *Technology System* section. The first 2 sessions served as introductory sessions in which the research assistant could provide guidance any time the participants showed signs of hesitation or difficulty. During the following (regular intervention) sessions, no research assistant’s guidance was available except if a participant asked for it.

### Data Recording

Data recording concerned (1) the number of task steps performed correctly (ie, in line with the step descriptions and independent of the research assistant’s guidance) within the sessions and (2) the length of the sessions. Data were recorded by the research assistants responsible for the implementation of the sessions. Interrater agreement was assessed by having a reliability observer record the participants’ performance of the task steps and the sessions’ length in 21% to 23% of the participants’ sessions. The percentage of agreement (calculated by dividing the number of sessions in which the 2 raters reported the same number of correct steps and session lengths differing by less than 1.5 minutes by the total number of sessions in which agreement was checked, and multiplying by 100%) ranged between 91 and 100% across participants.

## Results

**Figure 3:**
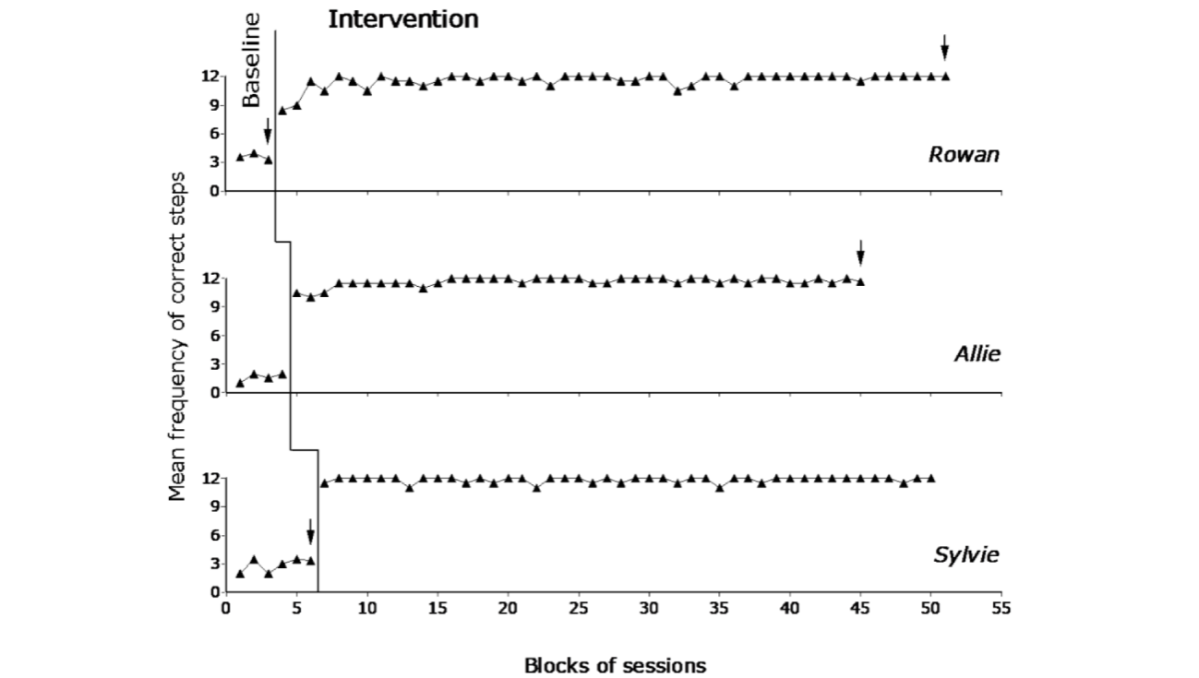
The 3 graphs report the baseline and intervention data for Rowan, Allie, and Sylvie. Each data point represents the mean frequency of correct steps over a block of 2 sessions. Blocks of 3 sessions are marked with an arrow.

**Figure 4:**
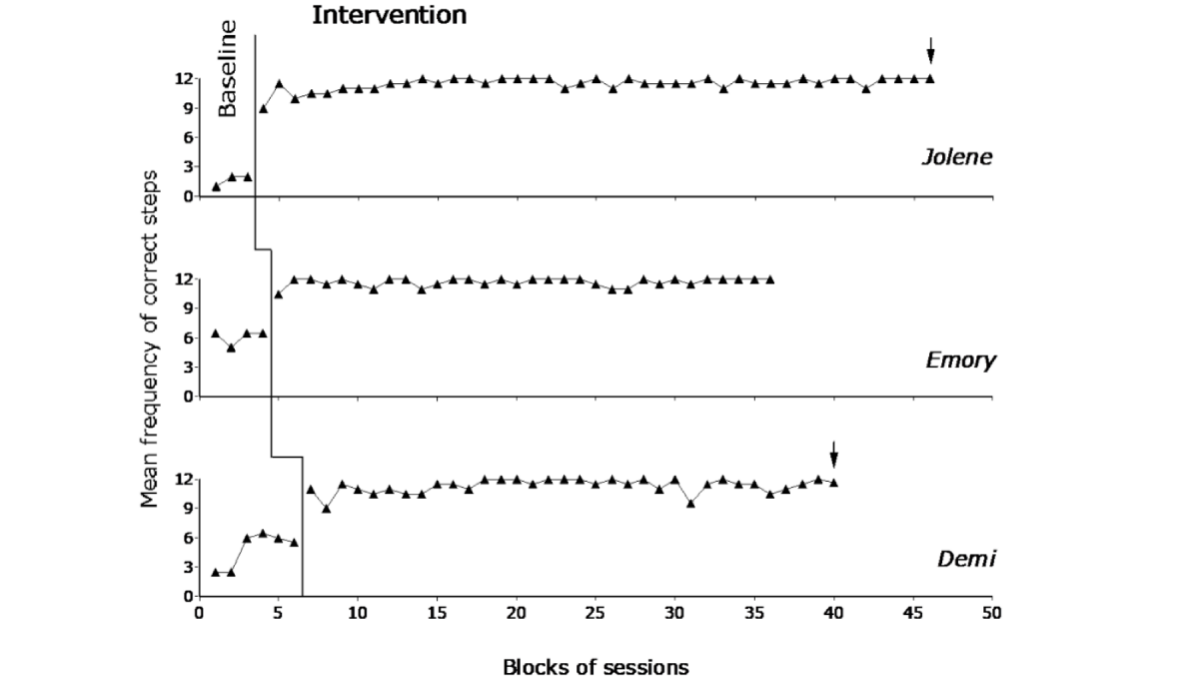
The 3 graphs report the baseline and intervention data for Jolene, Emory, and Demi. Data are plotted as in Figure 3.

Figures 3 and 4 report the baseline and intervention data for the first group of participants (ie, Rowan, Allie, and Sylvie) and the second group of participants (ie, Jolene, Emory, and Demi), respectively. The black triangles represent mean frequencies of correct task steps over blocks of 2 sessions. Occasional blocks with 3 sessions (at the end of the phases) are marked with an arrow. The figures do not report the 2 introductory sessions carried out at the start of the intervention phase.

During the pretest, the participants’ frequency of correct task steps per session was (virtually) zero. Indeed, they could carry out a single step (not necessarily involved in the task presented) or remain inactive. All sessions were interrupted after they had received guidance for 2 task steps. The mean session length was below 10 minutes for all participants.

During the baseline, the participants’ mean frequency of correct steps per session varied between about 1.5 (Allie) and 6 (Emory) out of the 12 steps available for each of the tasks. Such frequency reflected their inaccurate (unreliable) use of the tablet (ie, skipping step instructions or blocking the scrolling process and closing the instructions’ presentation) with the consequent omission of many task steps. The mean session length was about 6.5 (Jolene) to 14.5 (Emory) minutes. The mean length across participants was about 11.5 minutes.

During the intervention, the participants carried out the tasks successfully, and the mean frequency of task steps performed correctly per session varied between near 11.5 (Jolene and Demi) and above 11.5 (all other participants). The mean session length varied between about 15 (Demi) and 29.5 (Allie) minutes. The mean length across participants was about 19.5 minutes. The session length reported for pretest, baseline, and intervention always included the 2.5-minute preferred video shown at the end of the sessions. The large differences in the session length observed during the intervention (when the frequency of correct steps was similar across participants) mainly reflected differences in the participants’ performance speed. The Percentage of data points Exceeding the Median method showed indices of 1 for all participants (ie, all their intervention data points were higher than their median baseline frequency value) confirming the strong impact of the intervention with the technology system on their task performance.

## Discussion

### Principal Findings

The results suggest that the technology system used during the intervention was adequate to help the participants receive step instructions in a timely fashion and without the need to produce specific responses on the tablet. The participants’ high frequency of correct task steps and the stability of such frequency across the intervention phase suggest that the instruction process was suitable for them and that they had sufficient motivation to maintain their task performance over time [38-40]. In light of the above, a few considerations may be in order.

First, the new technology system seems to have the characteristics required to bypass the limitations of the two main instruction technology approaches typically used with people with intellectual and developmental disabilities, that is, the approach requiring the participants to seek the instructions through simple responses on the tablet or computer’s screen and the approach providing automatic presentation of the instructions, at preset time intervals [1,4]. Indeed, by avoiding the need for fine motor request responses, the new system can successfully help participants who, due to poor fine motor skills, would fail to benefit from the first approach. Moreover, by ensuring a timely presentation of the step instructions based on the participants’ walking to the tablet, the new system would avoid any reliance on prearranged instruction deliveries and related risks of instruction neglect in case of performance difficulties or slowness.

Second, the system can be flexible concerning the way the instructions are presented. As viewed in this study, for example, the system can be set to present the two instructions concerning each task step at successive times for people who can handle only one simple instruction at a time (people with poor working memory [41,42]). The system can also be set to present the two instructions of each step simultaneously for participants who are able to handle more complex instruction inputs. Technically, the system could also be set up to present the step instructions in small chunks with people who have a relatively high level of functioning or have become very familiar with the tasks on hand and no longer need an analytic step-by-step instruction process [43-46].

Third, the system can be easily used for supporting tasks that may change across days in terms of the steps included. The most direct and fast way to arrange the sequence of steps included in the task on any particular day is to provide the system with a sequence of numbers representing the codes for those steps [16]. To facilitate the use of the system by staff and caregivers who have limited familiarity with technology, the system could be fitted with a series of tasks and variations thereof that can be selected by writing their names or any other code used in storing them in the tablet memory.

Fourth, the use of a webcam to trigger the tablet to present instructions can be considered a rather simple technology solution [47-50]. The webcam is a small battery-powered device connected to the tablet via Bluetooth, a device that is much simpler and easier to operate than conventional motion sensors, such as the Philips Hue motion sensors [51]. Moreover, the webcam’s cost (about US $60) is largely affordable [52]. When using the system within a daily context, one would be advised to locate the webcam and the tablet in a room corner. This would minimize the risk that people sharing the room with the participants can accidentally interfere with the system’s functioning.

### Limitations and Future Research

The study presents 4 basic limitations, namely, the small number of participants, lack of generalization and maintenance data, lack of participants’ satisfaction data, and lack of social validation of the technology and its impact. The first limitation reflects the preliminary nature of the study, prevents one from making general statements about the findings reported, and underlines the need for new studies with additional participants [53-55]. The second limitation calls for new studies directed at (1) extending the number of sessions implemented and the intervention period to verify whether the intervention effects last and consolidate over time and (2) carrying out the sessions in different settings (provided these were familiar to the participants) to determine how extensively and profitably the system could be used within daily contexts [39,55-57].

The third limitation necessitates assessing how the participants perceive the intervention program. The assessment could consist of having the participants choose between the sessions with the system and other types of daily occupation. Large levels of preference for the sessions over other types of occupation would suggest participants’ satisfaction with the sessions [58-61]. The fourth limitation underlines the need for new studies to include staff and caregivers in the evaluation of the technology and its impact, as these personnel are finally responsible for applying the program and its technology in daily contexts. A practical way to include these personnel in the evaluation could involve (1) the personnel’s access to videos reporting the performance of different participants during intervention sessions and (2) the personnel’s rating of the videos on points such as the participants’ comfort during the sessions, the relevance of their task performance, and the overall acceptability and applicability of the intervention program [62,63].

### Conclusions

In conclusion, the results of this study suggest that the technology system used for the intervention program implemented with 6 participants was effective in helping them carry out fairly complex tasks independently and accurately. Although quite encouraging, these results are to be taken with caution, given the limitations of the study mentioned above. New studies should address those limitations and provide the evidence necessary to determine the applicability and impact of the present technology-aided program. New research may also assess the possibility of upgrading and optimizing the technology to facilitate and extend its use across settings and people.

## Data Availability

The data sets generated during and analyzed during this study are available from the corresponding author on reasonable request.

## References

[1] Desideri L, Lancioni G, Malavasi M, Gherardini A, Cesario L (2021). Step-instruction technology to help people with intellectual and other disabilities perform multistep tasks: a literature review. J Dev Phys Disabil.

[2] Golisz K, Waldman-Levi A, Swierat RP, Toglia J (2018). Adults with intellectual disabilities: case studies using everyday technology to support daily living skills. Br J Occup Ther.

[3] Lin M, Chiang M, Shih C, Li M (2018). Improving the occupational skills of students with intellectual disability by applying video prompting combined with dance pads. J Appl Res Intellect Disabil.

[4] Muharib R, Ledbetter-Cho K, Bross LA, Lang R, Hinson MD, Cilek RK (2022). Handheld Technology to Support Vocational Skills of Individuals with Intellectual and Developmental Disabilities in Authentic Settings: a Systematic Review. Rev J Autism Dev Disord.

[5] Randall KN, Johnson F, Adams SE, Kiss CW, Ryan JB (2020). Use of a iPhone task analysis application to increase employment-related chores for individuals with intellectual disabilities. J Spec Educ Technol.

[6] Dijkhuizen A, Hilgenkamp TI, Krijnen WP, van der Schans CP, Waninge A (2016). The impact of visual impairment on the ability to perform activities of daily living for persons with severe/profound intellectual disability. Res Dev Disabil.

[7] Lancioni GE, Singh NN, O'Reilly MF, Sigafoos J, Alberti G, Zimbaro C, Chiariello V (2017). Using smartphones to help people with intellectual and sensory disabilities perform daily activities. Front Public Health.

[8] Lancioni GE, O’Reilly MF, Sigafoos J, Alberti G, Tenerelli G, Ricci C, Marschik PB, Desideri L (2021). Tying the delivery of activity step instructions to step performance: evaluating a basic technology system with people with special needs. Adv Neurodev Disord.

[9] Heider AE, Cannella-Malone HI, Andzik NR (2019). Effects of self-directed video prompting on vocational task acquisition. CDTI.

[10] Johnson KR, Blaskowitz MG, Mahoney WJ (2023). Technology for adults with intellectual disability: secondary analysis of a scoping review. Can J Occup Ther.

[11] Park J, Bouck E, Duenas A (2019). The effect of video modeling and video prompting interventions on individuals with intellectual disability: a systematic literature review. J Spec Educ Technol.

[12] Resta E, Brunone L, D'Amico Fiora, Desideri L (2021). Evaluating a low-cost technology to enable people with intellectual disability or psychiatric disorders to initiate and perform functional daily activities. Int J Environ Res Public Health.

[13] Bigby C, Beadle-Brown Julie (2018). Improving quality of life outcomes in supported accommodation for people with intellectual disability: what makes a difference?. J Appl Res Intellect Disabil.

[14] Cummins RA (2020). Quality of life of adults with an intellectual disability. Curr Dev Disord Rep.

[15] Fekete C, Siegrist J, Post MWM, Brinkhof MWG, SwiSCI Study Group (2019). Productive activities, mental health and quality of life in disability: exploring the role enhancement and the role strain hypotheses. BMC Psychol.

[16] Lancioni GE, Singh NN, O'Reilly MF, Sigafoos J, Alberti G, Del Gaudio V, Abbatantuono C, Taurisano P, Desideri L (2022). People with intellectual and sensory disabilities can independently start and perform functional daily activities with the support of simple technology. PLoS One.

[17] Mechling LC, Gast DL, Seid NH (2010). Evaluation of a personal digital assistant as a self-prompting device for increasing multi-step task completion by students with moderate intellectual disabilities. Educ Train Autism Dev Disabil.

[18] Oh A, Gan S, Boscardin WJ, Allison TA, Barnes DE, Covinsky KE, Smith AK (2021). Engagement in meaningful activities among older adults with disability, dementia, and depression. JAMA Intern Med.

[19] Smith EM, Huff S, Wescott H, Daniel R, Ebuenyi ID, O'Donnell Joan, Maalim M, Zhang W, Khasnabis C, MacLachlan M (2024). Assistive technologies are central to the realization of the Convention on the Rights of Persons with Disabilities. Disabil Rehabil Assist Technol.

[20] Fernández-Batanero JM, Montenegro-Rueda M, Fernández-Cerero J, García-Martínez I (2022). Assistive technology for the inclusion of students with disabilities: a systematic review. Education Tech Research Dev.

[21] Khanlou N, Khan A, Vazquez LM, Zangeneh M (2021). Digital literacy, access to technology and inclusion for young adults with developmental disabilities. J Dev Phys Disabil.

[22] Collins JC, Ryan JB, Katsiyannis A, Yell M, Barrett DE (2014). Use of portable electronic assistive technology to improve independent job performance of young adults with intellectual disability. J Spec Educ Technol.

[23] Cullen JM, Alber-Morgan SR, Simmons-Reed EA, Izzo MV (2017). Effects of self-directed video prompting using iPads on the vocational task completion of young adults with intellectual and developmental disabilities. JVR.

[24] Cullen JM, Simmons‐Reed EA, Weaver L (2017). Using 21st century video prompting technology to facilitate the independence of individuals with intellectual and developmental disabilities. Psychology in the Schools.

[25] Lancioni Ge, Singh N, O′Reilly M, Sigafoos J, Alberti G, Boccasini A, Perilli V, Lang R (2014). A Computer-aided program regulating the presentation of visual instructions to support activity performance in persons with multiple disabilities. J Dev Phys Disabil.

[26] Lancioni GE, Singh NN, O'Reilly MF, Sigafoos J, Alberti G, Chiariello V, Carrella L (2020). Everyday technology to support leisure and daily activities in people with intellectual and other disabilities. Dev Neurorehabil.

[27] Ivey AN, Mechling LC, Spencer GP (2015). Use of a proximity sensor switch for "hands free" operation of computer-based video prompting by young adults with moderate intellectual disability. Educ Train Autism Dev Disabil.

[28] Mishra S, Laplante-Lévesque Ariane, Barbareschi G, Witte LD, Abdi S, Spann A, Khasnabis C, Allen M (2024). Assistive technology needs, access and coverage, and related barriers and facilitators in the WHO European region: a scoping review. Disabil Rehabil Assist Technol.

[29] Mihailidis A, Melonis M, Keyfitz R, Lanning M, Van Vuuren S, Bodine C (2016). A nonlinear contextually aware prompting system (N-CAPS) to assist workers with intellectual and developmental disabilities to perform factory assembly tasks: system overview and pilot testing. Disabil Rehabil Assist Technol.

[30] Balboni G, Belacchi C, Bonichini S, Coscarelli A (2016). Vineland-II. Vineland Adaptive Behavior Scales Second Edition. Standardizzazione Italiana.

[31] Sparrow S, Cicchetti D, Balla D (2005). Vineland Adaptive Behavior Scales.

[32] DEATTI.

[33] Lancioni GE, Desideri L, Singh NN, Sigafoos J, O'Reilly Mark F (2022). A commentary on standards for single-case experimental studies. Int J Dev Disabil.

[34] Ledford J, Gast D (2018). Single Case Research Methodology: Applications in Special Education and Behavioral Sciences.

[35] Strain P, Fox L, Barton EE (2021). On expanding the definition and use of procedural fidelity. RPSD.

[36] Ma H (2006). An alternative method for quantitative synthesis of single-subject researches: percentage of data points exceeding the median. Behav Modif.

[37] Parker RI, Vannest KJ, Davis JL (2011). Effect size in single-case research: a review of nine nonoverlap techniques. Behav Modif.

[38] Kocman A, Weber G (2018). Job satisfaction, quality of work life and work motivation in employees with intellectual disability: a systematic review. J Appl Res Intellect Disabil.

[39] Pierce W, Cheney C (2017). Behavior Analysis and Learning. Sixth Edition.

[40] Federici S, Scherer M (2017). Assistive Technology Assessment Handbook. Second Edition.

[41] Lifshitz H, Kilberg E, Vakil E (2016). Working memory studies among individuals with intellectual disability: an integrative research review. Res Dev Disabil.

[42] Vicari S, Costanzo F, Menghini D (2016). Memory and learning in intellectual disability. Int Rev Res Dev Disabil.

[43] Lancioni GE, Singh NN, O’Reilly MF, Sigafoos J (2024). Possible assistive technology solutions for people with moderate to severe/profound intellectual and multiple disabilities: considerations on their function and long-term role. Int J Dev Disabil.

[44] Shepley SB (2017). Self-instructing with mobile technology: considerations and applications to increase independence. Teach Except Child.

[45] Sigafoos J, O’Reilly M, Cannella H, Edrisinha C, de la Cruz B, Upadhyaya M, Lancioni GE, Hundley A, Andrews A, Garver C, Young D (2006). Evaluation of a video prompting and fading procedure for teaching dish washing skills to adults with developmental disabilities. J Behav Educ.

[46] Wu P, Cannella-Malone HI, Wheaton JE, Tullis CA (2014). Using video prompting with different fading procedures to teach daily living skills. Focus Autism Other Dev Disabl.

[47] Boot FH, Dinsmore J, Khasnabis C, MacLachlan M (2017). Intellectual disability and assistive technology: opening the GATE wider. Front Public Health.

[48] Borg J, Winberg M, Eide AH, Calvo I, Khasnabis C, Zhang W (2023). On the relation between assistive technology system elements and access to assistive products based on 20 country surveys. Healthcare (Basel).

[49] Botelho FHF (2021). Accessibility to digital technology: virtual barriers, real opportunities. Assist Technol.

[50] Horton S (2021). Empathy cannot sustain action in technology accessibility. Front Comput Sci.

[51] Lancioni GE, Singh NN, O'Reilly MF, Sigafoos J, Alberti G, Chiariello V, Campodonico F, Desideri L (2021). Technology-aided spatial cues, instructions, and preferred stimulation for supporting people with intellectual and visual disabilities in their occupational engagement and mobility: usability study. JMIR Rehabil Assist Technol.

[52] Abdi S, Kitsara I, Hawley MS, de Witte LP (2021). Emerging technologies and their potential for generating new assistive technologies. Assist Technol.

[53] Kazdin A (2011). Single-Case Research Designs: Methods for Clinical and Applied Settings. Second Edition.

[54] Locey ML (2020). The evolution of behavior analysis: toward a replication crisis?. Perspect Behav Sci.

[55] Walker SG, Carr JE (2021). Generality of findings from single-case designs: it's not all about the " N". Behav Anal Pract.

[56] Pennington B, Simacek J, McComas J, McMaster K, Elmquist M (2019). Maintenance and generalization in functional behavior assessment/behavior intervention plan literature. J Behav Educ.

[57] Smith KA, Ayres KA, Alexander J, Ledford JR, Shepley C, Shepley Sally B (2016). Initiation and generalization of self-instructional skills in adolescents with autism and intellectual disability. J Autism Dev Disord.

[58] Carney T, Then S, Bigby C, Wiesel I, Douglas J, Smith E (2019). Realising ‘will, preferences and rights’: reconciling differences on best practice support for decision-making?. Griffith Law Review.

[59] Ninci J, Gerow S, Rispoli M, Boles M (2017). Systematic review of vocational preferences on behavioral outcomes of individuals with disabilities. J Dev Phys Disabil.

[60] Tullis C, Cannella-Malone H, Basbigill A, Yeager A, Fleming C, Payne D, Wu P (2011). Review of the choice and preference assessment literature for individuals with severe to profound disabilities. Educ Train Autism Dev Disabil.

[61] Wehmeyer ML (2020). The importance of self-determination to the quality of life of people with intellectual disability: a perspective. Int J Environ Res Public Health.

[62] Stasolla F, Caffò AO, Perilli V, Albano V (2019). Experimental examination and social validation of a microswitch intervention to improve choice-making and activity engagement for six girls with Rett syndrome. Dev Neurorehabil.

[63] Worthen D, Luiselli JK (2019). Comparative effects and social validation of support strategies to promote mindfulness practices among high school students. Child Fam Behav Ther.

